# The Effects of Larval Cryopreservation on the Epigenetics of the Pacific Oyster *Crassostrea gigas*

**DOI:** 10.3390/ijms242417262

**Published:** 2023-12-08

**Authors:** Yibing Liu, Lisui Bao, Sarah R. Catalano, Xiaochen Zhu, Xiaoxu Li

**Affiliations:** 1 Fisheries College, Ocean University of China, Qingdao 266003, China; liuyibing@ouc.edu.cn; 2 Institute of Evolution & Marine Biodiversity, Ocean University of China, Qingdao 266003, China; baolisui@ouc.edu.cn; 3 Aquatic Sciences Centre, South Australian Research and Development Institute, Adelaide 5024, Australia; sarah.catalano@sa.gov.au; 4 College of Science and Engineering, Flinders University, Adelaide 5042, Australia; asawinter2003@hotmail.com

**Keywords:** Pacific oyster, larval cryopreservation, DNA methylation, histone modification, gene expression

## Abstract

High mortalities and highly variable results during the subsequent development of post-thaw larvae have been widely considered as key issues restricting the application of cryopreservation techniques to support genetic improvement programs and hatchery production in farmed marine bivalve species. To date, few studies have been undertaken to investigate the effects of cryodamage at the molecular level in bivalves. This study is the first to evaluate the effect of larval cryopreservation on the epigenetics of the resultant progenies of the Pacific oyster *Crassostrea gigas*. The results show that the level of DNA methylation was significantly (*p* < 0.05) higher and lower than that of the control when the trochophore larvae were revived and when they developed to D-stage larvae (day 1 post-fertilization), respectively, but the level returned to the control level from day 8 post-fertilization onwards. The expression of the epigenetic regulator genes *DNMT3b*, *MeCP*2, *JmjCA*, *KDM2* and *OSA* changed significantly (*p* < 0.05) when the trochophore larvae were thawed, and then they reverted to the control levels at the D- and later larval developmental stages. However, the expression of other epigenetic regulator genes, namely, *MBD2*, *DNMT1*, *CXXC1* and *JmjD6*, did not change at any post-thaw larval developmental stage. For the newly thawed trochophore larvae, the amount of methylated H3K4Me1 and H3K27Me1 significantly changed, and the expression of all *Jumonji* orthologs, except that of *Jumonji5,* significantly (*p* < 0.05) decreased. These epigenetic results agree with the data collected on larval performances (e.g., survival rate), suggesting that the effect period of the published cryopreservation technique on post-thaw larvae is short in *C. gigas*.

## 1. Introduction

Mollusk farming is the second largest aquaculture sector in the world by both quantity and value [1], with the Pacific oyster *Crassostrea gigas* being one of the leading species by quantity and being mainly farmed in Australia, New Zealand, the USA, and European and Asian countries [1,2]. The spats produced in hatcheries have become the preferential source for the industry [2] due to their capacities to reliably supply high-quality spats for on-growing and to establish genetic improvement programs for the long-term sustainable development of the industry [2]. Today, selective breeding programs have been established, and selective bred broodstock have been used in *C. gigas* commercial production in many countries [2,3]. Nevertheless, due to the lack of reliable techniques to safely protect the superior genetic resources established, these programs could be jeopardized by unpredictable occurrences, such as disease outbreaks, which have been reported in *C. gigas* in many countries [4,5,6,7]. Larval cryopreservation has been widely acknowledged as a safe and effective technique for this purpose in aquatic species because the preservation of both parental genetics has been compromised by unsuccessful attempts to cryopreserve oocytes [8]. This technique could also be a powerful tool to assist in hatchery production without the limitation of seasons. However, challenges have been evident in developing this technique in marine bivalve species, with spats from post-thaw larvae being produced in only three species, including *C. gigas* [8,9,10]. In *C. gigas*, it is currently impractical to use the larval cryopreservation technique in commercial settings due to (a) the lack of information on the cryo-impacts on the genetics of the resulting progeny and (b) the high mortalities and extremely variable results from cryopreserved larvae, with the highest spat rate of ~5% being achieved by Liu et al. [11]. In order to further improve both genetic knowledge and techniques, it is paramount to investigate the cryodamage to larvae.

Parameters such as larval performance (particularly the survival rate), morphology and organogenesis have been used to develop or improve the larval cryopreservation technique for marine bivalve species, but few studies have investigated the potential cryodamage that occurs at the molecular level [12,13,14]. Recently, studies on epigenetics against cryopreservation have attracted attention, with epigenetics being used as a tool to understand the mechanism of cryodamage [15,16]. Usually, DNA methylation and histone modification are referred to as epigenetic mechanisms that influence gene expression [17,18]. DNA methylation is important for cell-type differentiation and embryonic development [18]. Histone modification is also critical for embryonic development, with the methylation of histone residues (a particular histone modification) playing an essential role in the regulation of gene transcription, promoting the activation or silencing of genes [19]. Both mechanisms have been found to be sensitive to temperature changes [18,20].

In aquatic species, the development of embryo and larval cryopreservation techniques has been very challenging mainly due to the large size, high lipid content and complex structures of the embryo and larvae, and the technique has only been successfully achieved recently in a handful of species, mainly in bivalves [8,9]. The programmable cryopreservation technique is the method that has been reported so far in bivalves, where a reduction in temperature during larval cryopreservation is managed by a controlled-rate freezer to a predetermined subzero temperature followed by plunging into liquid nitrogen for storage at −196 °C [8,9,10,11]. This extremely low-temperature condition and the temperatures experienced during the cryopreservation process could influence epigenetic mechanisms. In animals, gamete and embryo cryopreservation has been linked to epigenetic modifications; it is specifically associated with reduced cellular survival and impaired embryonic development [21]. For example, Maldonado et al. [17] reported that the tri-methylation profiles of lysines 4 and 27 of histone H3 were affected by cryopreservation, with the levels of H3K4me3 and H3K27me3 being significantly decreased (20% lower) and increased (nearly 2-fold higher), respectively, in post-thaw bovine blastocysts in comparison with their fresh counterparts. These histone modifications were suggested as being the reason for the poor pregnancy rates in bovines when cryopreserved blastocysts were used [17]. In mice, the intensity of H3K9 of post-thaw oocytes was significantly increased after 2 h of culture, which was attributed to the compromised oocyte quality after cryopreservation [22]. Yan et al. [22] therefore suggested that histone modification could be used as a marker to optimize the oocyte cryopreservation protocol in mice. In dromedary camels, Moulavi et al. [23] found that DNA methylation was significantly changed in two-cell embryos derived from post-thaw oocytes, with levels returning to the control state at the blastocyst stage.

In *C. gigas*, DNA methylation and histone modification have been found to be essential for the development of embryos and larvae [24,25,26,27]. Moreover, *Jumonji* genes have been implicated in epigenetically regulating the development of animals and displayed a ubiquitous expression during the embryonic and larval development of *C. gigas* [28]. To date, nine *Jumonji* orthologs have been reported in this species [28]. In addition, Riviere et al. [29] considered that this species could be used as a model species to elucidate the effects of epigenetics on larval development in Lophotrochozoa. Currently, the effects of cryopreservation on the epigenetics of larvae have not been investigated in marine bivalve species. Therefore, in this study, *C. gigas* was used as a model species for marine bivalve species to understand the effects of the published cryopreservation technique on DNA methylation and the expression of epigenetic regulator genes from cryopreserved trochophore larvae to their further development into spats. In addition, the histone modifications and mRNA expressions of *Jumonji* orthologs were assessed in post-thaw trochophore larvae only.

## 2. Results

### 2.1. Comparison of Larval Performance in Control and Treatment Groups across Developmental Stages in C. gigas

The treatment group (cryopreserved larvae) was characterized as having a significantly lower survival rate (*p* < 0.05), but there were no differences in shell length (*p* > 0.05) across all larval developmental stages compared with the control group (Table 1). Conversely, the relative survival rate of the treatment group was significantly lower than that of the control group (*p* < 0.05) only at the umbo larval stage (Table 1).

### 2.2. Comparison of DNA Methylation and Gene Expressions of Epigenetic Regulators in Control and Treatment Groups across Developmental Stages in C. gigas

**Figure 1:**
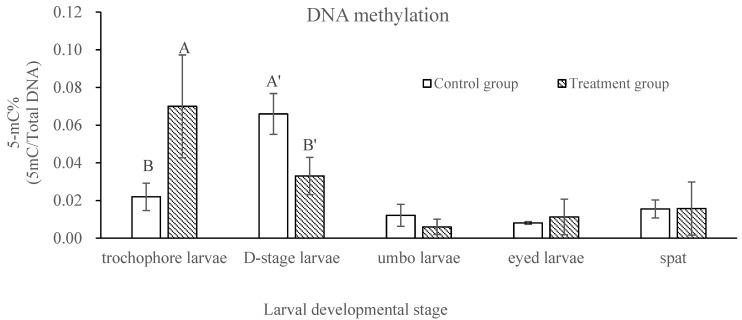
Comparison of DNA methylation between control and treatment groups at different larval developmental stages in *C. gigas*, *n* = 3. Different letters at the same developmental stage indicate a significant difference, *p* < 0.05.

**Figure 2:**
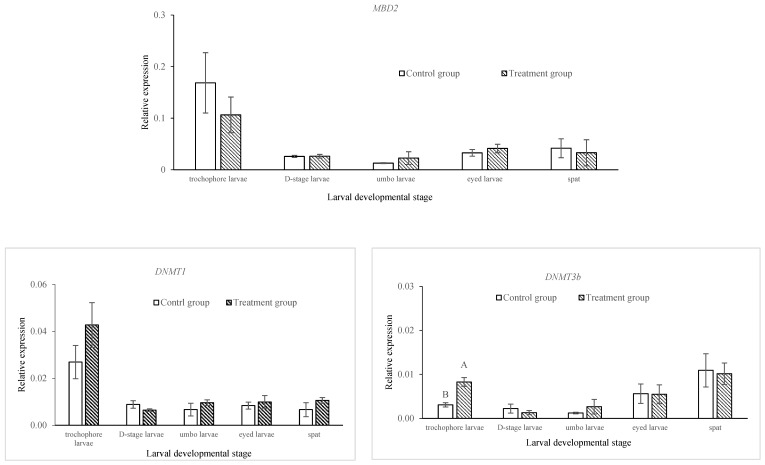

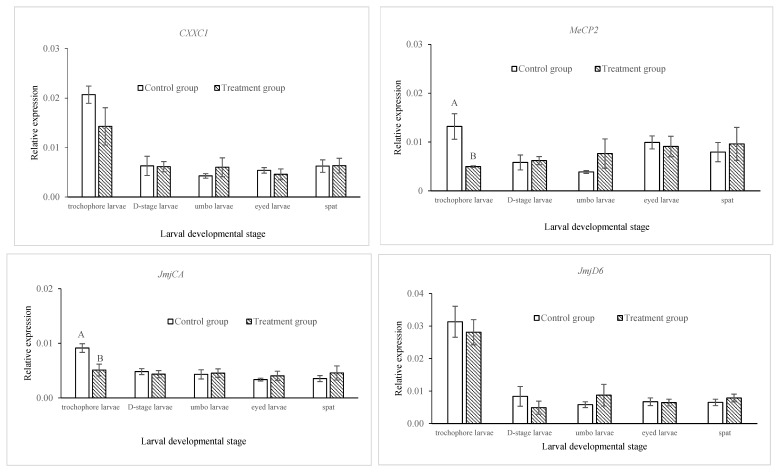

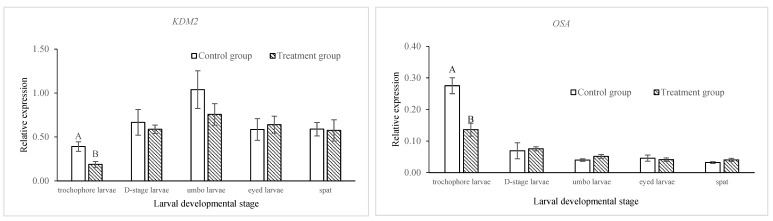
Comparison of the expression of epigenetic regulator genes between control and treatment groups at different larval developmental stages in *C*. *gigas*, *n* = 3. Different letters at the same developmental stage indicate a significant difference, *p* < 0.05.

At the post-thaw trochophore stage, the level of DNA methylation in the treatment group was significantly higher than that in the control group (*p* < 0.05), and then it dropped to become significantly lower (*p* < 0.05) at the D-stage, with no difference (*p* > 0.05) in the three remaining developmental stages (Figure 1). For the epigenetic regulator genes, significantly different levels of expression were observed only at the trochophore stage for the DNA methylation machinery genes *DNMT3b* and *MeCP*2 and for the histone modifiers *JmjCA*, *KDM2* and *OSA*, with a higher expression for *DNMT3b* and a lower expression for *MeCP*2, *JmjCA*, *KDM2* and *OSA* in the treatment group than in the control group (*p* < 0.05; Figure 2). There was no significant difference in the expressions of the *MBD2*, *DNMT1*, *CXXC1* (all DNA methylation machinery) or *JmjD6* (histone modifier) genes between the control and treatment groups at all larval developmental stages (*p* > 0.05; Figure 2).

### 2.3. Comparison of the Expression of Jumonji Orthologs between Control and Treatment Groups in Trochophore Larvae

**Figure 3:**
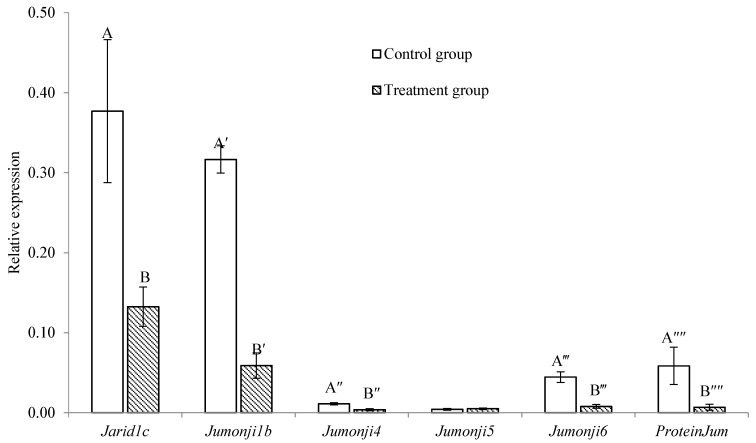
Comparison of the expression of *Jumonji* orthologs in trochophore larvae between the control and treatment groups in *C. gigas*, *n* = 3. Different letters at the same gene indicate a significant difference, *p* < 0.05.

The mRNA expression of all *Jumonji* orthologue genes was significantly lower (*p* < 0.05; Figure 3) in the post-thaw trochophore larvae than in the control except for that of the *Jumonji 5* gene (*p* > 0.05; Figure 3).

### 2.4. Comparison of the Mono-, Di- and Tri-Methylation Profiles between Control and Treatment Groups of Histone 3 at Lysines 4, 9 and 27 in Trochophore Larvae

**Figure 4:**
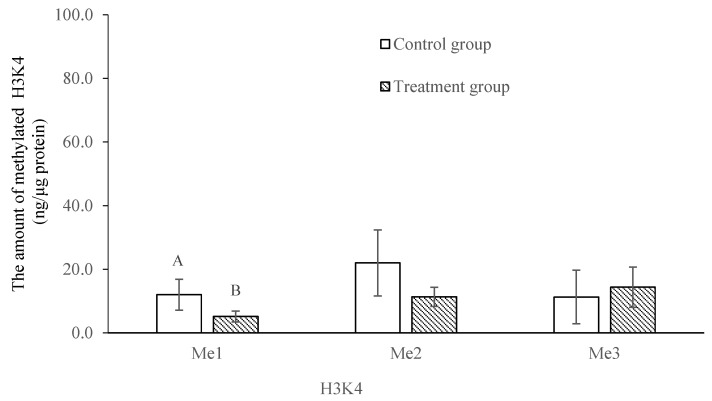
Comparison of the mono-, di- and tri-methylation profiles of lysine 4 of histone 3 in trochophore larvae between control and treatment groups in *C. gigas*, *n* = 3. Different letters at the same methylation profile indicate a significant difference, *p* < 0.05.

**Figure 5:**
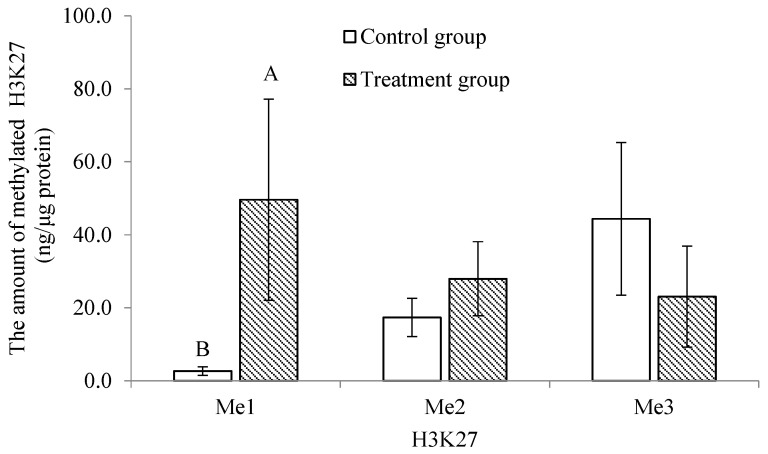
Comparison of the mono-, di- and tri-methylation profiles of lysine 27 of histone 3 in trochophore larvae between control and treatment groups in *C. gigas*, *n* = 3. Different letters at the same methylation gene indicate a significant difference, *p* < 0.05.

**Figure 6:**
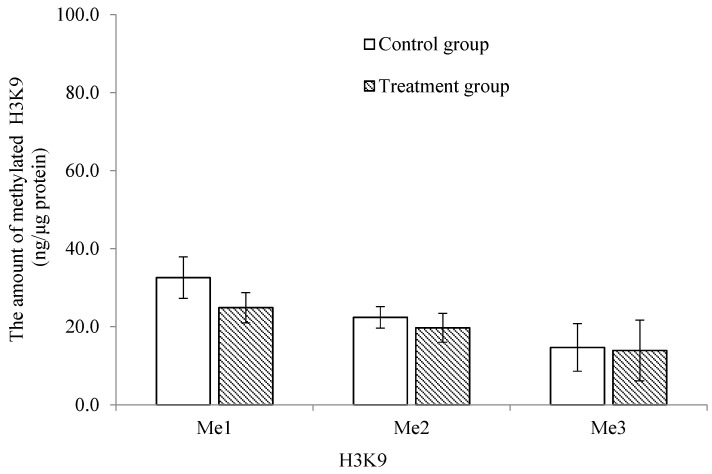
Comparison of the mono-, di- and tri-methylation profiles of lysine 9 of histone 3 in trochophore larvae between control and treatment groups in *C. gigas*, *n* = 3.

The methylation profile of the trochophore larvae was significantly lower and higher (*p* < 0.05) in the treatment group for the mono-methylation of histone 3 at lysine 4 (H3K4) and lysine 27 (H3K27), respectively (Figure 4 and Figure 5). There was no significant difference in the di- and tri-methylation profiles of H3K4 and H3K27 or in the mono-, di- and tri- methylation profiles of histone 3 at lysine 9 (H3K9) between the treatment and the control groups (*p* > 0.05; Figure 4, Figure 5 and Figure 6).

## 3. Discussion

In marine bivalve aquaculture, the high mortality, the extremely variable outcome of post-thaw larvae and the knowledge gaps of the cryo-impact on genetics are widely accepted as key issues limiting the application of the larval cryopreservation technique. Although a few studies have investigated the mechanisms of cryodamage to larvae, there has been little research undertaken at the molecular level [8]. Our study investigated the performance of the post-thaw larvae of *C. gigas* along with the molecular changes that occur (e.g., epigenetics) as a result of cryopreservation. This included (1) examining the levels of DNA methylation from trochophore larvae to spats; (2) quantifying the mRNA expression of DNA methylation machinery and histone modifiers (from trochophore larvae to spats) and *Jumonji* orthologs (trochophore larvae only); and (3) quantifying the mono-, di- and tri-methylated histone H3 at lysines 4, 9 and 27 (H3K4, H3K9 and H3K27) for trochophore larvae. To the best of our knowledge, this is the first study to assess the effects of cryopreservation on the epigenetics of larvae in marine bivalve species.

Epigenetics is referred to as the heritable modification of gene expression without the modification of the DNA sequence, and it encompasses various molecular mechanisms, including DNA methylation and histone modification [28]. In animals, DNA methylation primarily refers to the enzymatic addition of a methyl group to position 5 of cytosine residues to form 5-methylcytosine, which occurs almost exclusively at CpG dinucleotides [18,29]. DNA methylation influences cell differentiation and is important in the genome defense against transposable element activity, the maintenance of parental imprints and X chromosome inactivation [18]. In mammals, DNA methylation generally occurs at the promoter regions with the hyper-methylation of promoters having the potential to inhibit the initiation of transcription [30]. On the contrary, for invertebrates, DNA methylation patterns are primarily intragenic, and their genomes do not show differentially methylated gene promoters [31,32]. For aquaculture species, DNA methylation is the most well-studied epigenetic marker, with it being highly dynamic at precise locations during development [18].

The *C. gigas* genome, like that of other invertebrates, also displays intragenic DNA methylation [26]. By analyzing the differentially methylated regions (DMRs), Riviere et al. [27] separated the four main developmental phases in *C. gigas*, namely, oocytes, 2–8 cells, mid-larvae and spats, into three main developmental steps: (1) cleavage (C step), (2) gastrulation and organogenesis (I step, intermediate), and (3) metamorphosis (M step). The mid-larval developmental phase includes morula, blastula, gastrula, trochophore and D-stage larvae due to their minor differences in DMRs and the methylation profiles of individual features [27]. Riviere et al. [24] also reported that the morphological alteration and malformation of organogenesis establishment might result from gene transcription alterations induced by the dramatic changes in DNA methylation. In comparison to gastrulae and D-stage larvae, it was found that DNA methylation occurred more in trochophore larvae [24]. In the current study, significant changes in the level of DNA methylation were observed in the newly thawed trochophore larvae. These changes were further maintained until the larvae reached the D-stage, and they returned to the control level at the remaining three larval developmental stages in the control and treatment groups. This could be one of the key reasons for the significantly lower relative survival rate at this stage in the treatment group in this study. This also suggests that the larval cryopreservation technique could be effectively applied in hatchery production if the purge of unhealthy post-thaw larvae over the period until the umbonal stage is considered. However, as DNA methylation could turn genes off [33], caution should be taken when the post-thaw larvae are used for molecular investigations on the expression/improvement of traits of economic importance and questions related to early larval development in genetic improvement programs or studies. A similar phenomenon has also been reported in other species. For example, Chen et al. [34] and Liang et al. [35] found that the development potential of post-thaw bovine and mouse oocytes was partially compromised due to the level of DNA methylation being significantly lower than that of the controls in the initial development up to eight cells.

DNA methylation can also influence the chromatin compaction via methyl-DNA binding domain (MBD) proteins [24]. DNA methyltransferases (DNMTs), such as DNMT1 and DNMT3b, play important roles in the establishment and maintenance of DNA methylation patterns [36]. According to the definition by Riviere et al. [24], the epigenetic regulators evaluated in this study, the orthologs of MBD (CXXC-1, MBD2 and MeCP2) and DNMTs (DNMT1 and DNMT3b), belong to DNA methylation machinery. DNMT1 is a maintenance enzyme and is responsible for copying pre-existing DNA methylation patterns to a new strand during mitosis, whereas DNMT3b catalyzes de novo methylation [24]. Results from studies undertaken in mammals indicate that the MBD2, MeCP2 and CXXC-1 genes may mediate the downstream outcomes of DNA methylation, such as transcriptional regulation through chromatin remodeling [37]. Among the DNA methylation machinery genes investigated in this study, the expressions of *MeCP2* and *DNMT3b* significantly decreased and increased, respectively, in the post-thaw trochophore larvae. The increase in the expression of *DNMT3b* might be due to the increase in DNA methylation at this developmental stage in the treatment group. This result agrees with the findings by Riviere et al. [24], who found that the increase in DNA methylation at the trochophore stage matched the peak of *DNMT3b* expression.

According to Riviere et al. [24], the jmjCA, jmjD6, OSA and KDM2 genes investigated in this study are categorized as histone modifiers. jmjCA, jmjD6 and KDM2 are conserved domains [38,39,40], while OSA is a DNA-binding domain [41]. In the current study, the expressions of three of the four histone modifier genes investigated (i.e., JmjCA, KDM2 and OSA) were significantly lower in the treatment group than in the control group only at the trochophore stage. As these genes are important for a broad range of cellular processes, such as cell proliferation, differentiation and senescence [42,43,44], the observed result of a change in expression recorded for the post-thaw trochophore larvae could indicate a breakdown in functionality and disruption to normal cellular processes, leading to the low rate of D-stage larvae that developed from them.

In *C. gigas*, the orthologs of *Jumonji* have been characterized and considered to be critical for putative histone demethylase activities, leading to them being considered at least partly responsible for the changes in methyl-histone levels [20,28]. In our study, the expressions of all *Jumonji* orthologue genes, except that of *Jumonji5*, were significantly lower in the post-thaw trochophore larvae. As the proteins in the *Jumonji* family have the capability to regulate gene expression and control development [20,28,41], the changes observed in expression after cryopreservation have the potential to negatively influence the development of trochophore larvae, which could be a contributing factor toward the high mortality rate of post-thaw larvae.

Histone methylation plays an important role in regulating transcription, genome integrity and epigenetic inheritance, and it involves lysine residues, which serve as one of the most common acceptor sites of methylation marks [19,22]. Abnormal histone methylation patterns can alter the chromatin architecture and the accessibility of transcriptional machinery to DNA and influence the epigenetic memory system that regulates cell fate and identity [20,45]. Histone H3 is the primary site of histone methylation, with histone lysine methylation existing in a mono-, di- or tri-methylation state. Di- and tri-methylations at H3K4 are typically gene activating with H3K4me3 marking promoters [19]. H3K4me1 is an activating mark unique to enhancers [46]. H3K9 and H3K27 methylations are generally gene repressive, but they serve unique functions [47,48]. In *C. gigas*, Fellous et al. [25] found that the mono-methylation of H3K4 and H3K27 was stable at the trochophore stage. In the present study, the post-thaw trochophore larvae had a significantly lower mono-methylation of H3K4. This result may indicate that the activating mark was restricted, which could silence pluripotency genes and delay cell proliferation [20]. On the contrary, the mono-methylation of H3K27 was significantly higher at this stage. This phenomenon may be caused by the significant decrease in the jmjC histone demethylases, resulting in the compromise of larval physiology [25]. The three methylation states of H3K9 were not significantly altered in the post-thaw trochophore larvae. This result differs from that of Spinaci et al. [49], who reported that the cryopreservation technique altered the methylation of H3K9 in pig oocytes, potentially resulting in an aberrant epigenetic presentation of female chromatin to the fertilizing event and a reduction in their developmental competence.

## 4. Materials and Methods

### 4.1. Larvae Preparation

The *C. gigas* broodstock were supplied by a commercial farm at Coffin Bay, Adelaide, Australia, and transported in a refrigerated container to the South Australian Research and Development Institute (SARDI, Adelaide, Australia). The methods for broodstock maintenance, spawning induction and fertilization were the same as those described by Liu et al. [11]. Trochophore larvae at 18 h post-fertilization were collected, counted and diluted to 4 × 10^5^ individuals mL^−1^, and then they were divided into two tubes, one for the control group and the other for the treatment group. For the control group, a small portion of fresh trochophore larvae were transferred into a 2 mL vial and stored at −80 °C until required for further analysis. The remaining portion was cultured in a 20 L bucket and collected at D-stage larvae (day 1 post-fertilization (PF)), umbo larvae (day 8 PF), eyed larvae (day 22 PF) and spats (day 27 PF). For the treatment group, the trochophore larvae were cryopreserved following the protocol reported by Liu et al. [11]. Briefly, the trochophore larvae were mixed with 10% ethylene glycol + 5% Ficoll + 0.2% polyvinylpyrrolidone (final concentrations) for 10 min on ice. The mixtures were then transferred into 0.25 mL straws and maintained at 0 °C for 5 min in a programmable freezer. The straws were cooled at a rate of −1 °C/min from 0 to −10 °C and at −0.3 °C/min from −10 to −34 °C before being plunged into liquid nitrogen. The straws were thawed individually in a 28 °C water bath until the ice melted and then recovered in an 18 °C water bath. After thawing, a small portion of the post-thaw trochophore larvae was transferred into a 2 mL vial and stored at −80 °C for subsequent analysis, while the larger component was cultured for a subsequent assessment at the same developmental stages as the control group. Three replicates were assessed for the control and treatment groups. The methods used by Liu et al. [11,14] for larval culture, D-larvae rate (%) calculation, spat production, sample collection/assessment, survival rate (%) and relative survival rate (%) calculations at various developmental stages were followed.

### 4.2. Global DNA Methylation Quantification

The genomic DNA was extracted from the larvae at different developmental stages in both the control and treatment groups using a DNeasy Blood & Tissue Kit according to the manufacturer’s instructions. The methylated DNA was recognized by the 5-methylcytosine (5-mC) antibody and quantified through an ELISA-like reaction by using a MethylFlash™ Methylated DNA Quantification Kit (Epigentek, Farmingdale, NY, USA). The methylation percentage of each sample was calculated according to the slope of a standard curve, which was generated using the 100% methylated DNA standard.

### 4.3. Histone Extraction and Quantification

The histones were extracted from the fresh and post-thaw trochophore larvae using a Total Histone Extraction Kit (Epigentek). The trochophore larvae were harvested via centrifugation at 10,000 rpm for 5 min at 4 °C. Approximately 30 mg of larvae was re-suspended in 1 × pre-lysis buffer and disaggregated via bead beating. The homogenized mixture was transferred and centrifuged at 10,000 rpm for 1 min at 4 °C. After the removal of the supernatant, the larvae were re-suspended in a lysis buffer (at 3 × the volume of larvae) and incubated on ice for 30 min before being centrifuged at 12,000 rpm for 5 min at 4 °C; then, the supernatant fraction was transferred into a new vial. A balance-DTT buffer at 0.3 × the volume of the supernatant fraction was added immediately. The protein concentration was quantified using the Bradford method [20] with bovine serum albumin (BSA) used as the standard.

### 4.4. Histone Modification Quantification

The methylated histone H3 at lysines 4, 9 and 27 (H3K4, H3K9 and H3K27) was quantified from both the fresh and post-thaw trochophore larvae using the antibodies specific to mono-, di- and tri-methylated histone H3 (EpiQuik Global Pan-Methyl Histone (H3K4, H3K9 and H3K27) Quantification Kit (Fluorometric)). The mono-, di-, and tri-methyl H3K4, H3K9 and H3K27 were detected with a labeled detection antibody, which was followed by a fluorescent development reagent. The fluorescence was measured at 530 nm (excitation) and 590 nm (emission). The absolute amount of methylated H3 was calculated by using the following formula provided with the kit:

Amount (ng/mg protein) = RFU (sample − blank) × 1000/[Protein (μg) × slope],

where RFU is the relative fluorescence unit.

### 4.5. RNA Extraction and cDNA Synthesis

The total RNA was isolated from the larvae (~20 mg) at each developmental stage in the control and treatment groups using an RNeasy mini kit (Qiagen, Clayton, VIC, Australia) before being digested using a TURBO DNA-free™ Kit (Thermo Fisher Scientific, Waltham, MA, USA) to eliminate genomic DNA. The total RNA was purified and then measured on a NanoDrop 2000 (Thermo Fisher Scientific). Up to 5 μg of purified RNA was reverse-transcribed using a SuperScript™ III First-Strand Synthesis System (Thermo Fisher Scientific). The resulting cDNA was diluted, and the equivalent amount of 10 ng of purified RNA was used for gene expression.

### 4.6. Quantification of mRNA

The quantification of mRNA expression was performed using quantitative real-time PCR (qPCR). Three types of genes were selected: (1) DNA methylation machinery: *DNMT1*, *DNMT3b*, *MBD2*, *MeCP2* and *CXXC1*; (2) histone modifiers: *OSA*, *JmjCA*, *JmjD6* and *KDM2*; and (3) *Jumonji* orthologs: *Jarid 1c*, *Jumonji 1b*, *Jumonji 4*, *Jumonji 5*, *Jumonji 6* and *Protein Jumonji* [24,28]. The specific primers used for each gene were cited from published papers on the same species [24,28] and are listed in Table 2. Elongation-factor alpha (Efα; GenBank accession number BAD15289) was used as a reference gene for normalization purposes [28]. The expressions of the DNA methylation machinery and histone modifier genes were assessed across all larval developmental stages (from the trochophore larvae to spats), whereas the expressions of the *Jumonji* orthologs were assessed only at the trochophore stage. All qPCR reactions were performed using a StepOnePlus Real-Time PCR System (Applied Biosystems, Waltham, MA, USA). The reaction mixture contained 10 μL of PowerUp™ SYBR™ Green Master Mix, 1 μL of 10 μM forward and reverse primers, and 10 ng of cDNA template. An enzyme activation step of 2 min at 50 °C and a pre-incubation step of 2 min at 95 °C were included. cDNA amplification was carried out over 40 cycles under the following conditions: denaturation for 15 s at 95 °C, annealing for 1 min at 60 °C and a final melting gradient up to 95 °C using a ramp of 0.4 °C/s to check primer specificity. The relative abundance of genes was calculated using the 2^−∆*C*t^ method [14].

### 4.7. Statistical Analysis

The data were arcsine-transformed (D-stage larvae rate, survival and relative survival rates) before statistical analyses were performed using SPSS 22. A *t*-test was applied to compare the results between the control and treatment groups. Differences were considered statistically significant at *p* < 0.05. The results are presented as the mean ± standard deviation (SD).

## 5. Conclusions

In conclusion, both challenges and key knowledge gaps in larval cryopreservation have constrained the development and application of this technique in aquatic species. This study is the first investigation on the epigenetics of trochophore larvae and their resultant progenies in *C. gigas* aiming to understand and/or alleviate the challenges encountered in bivalve larval cryopreservation. The results indicate that the published cryopreservation technique affected the epigenetics of post-thaw trochophore larvae in the first few days until the umbonal stage. Therefore, future studies should focus on the mechanisms responsible for these observed changes and strategies that can be implemented to prevent the disruption of the epigenetic process in cryopreserved larvae, aiming to enhance survival and facilitate the broader application of this technique as an effective and safe management strategy for the long-term sustainability of the marine bivalve aquaculture industry. As epigenetics could influence gene expression, caution should be taken if cryopreserved larvae are used in molecular investigations within a short period after thawing.

## Figures and Tables

**Table 1:** Comparison of larval performance at each developmental stage between control and treatment groups in *C. gigas*.

Days Post-Fertilization	Survival Rate (%)		Relative Survival Rate (%)		Shell Length (µm)	
	Control Group	Treatment Group	Control Group	Treatment Group	Control Group	Treatment Group
Day 1 (D-stage larvae)	81.7 ± 5.8	61.3 ± 3.1 *			81.8 ± 3.5	80.2 ± 2.4
Day 8 (umbo larvae)	51.3 ± 7.2	20.3 ± 4.5 *	62.6 ± 4.6	33.3 ± 7.7 *	105.8 ± 3.2	103.8 ± 4.2
Day 22 (eyed larvae)	31.7 ± 9.3	7.7 ± 3.8 *	63.8 ± 25.5	38.4 ± 19.0	277.0 ± 12.3	272.0 ± 7.8
Day 27 (spat)	15.7 ± 6.0	4.7 ± 1.2 *	49.4 ± 10.9	65.6 ± 15.0	465.9 ± 30.0	455.7 ± 40.9

The asterisk indicates a significant difference (*p* < 0.05) between the control and treatment groups.

**Table 2:** Forward and reverse primer sequences of expressed genes between the control and treatment groups in *C. gigas*.

Gene	Forward Primer Sequence	Reverse Primer Sequence
DNA methylation machinery		
*DNMT1*	TTGGCAACATTCTGGACAAA	CGGTCTTCCATTCCAGTGAC
*DNMT3b*	TCTCTCAAGCAGGGGAGAAA	TGCTCTGGAAACCCAAAGAC
*MBD2*	TGACTTCCGCAGTGGTAGAA	ACTGTCGTGCCTCATTCCTC
*MeCP2*	ATGCAACCCTCAACCCAATA	GCCAAACTCATCGCCTGTAT
*CXXC1*	CGGCAAGATGCACAGTAGAA	CGGTTCATGATTGGTTGTGA
Histone modifier		
*OSA*	AACGAGATTGAGGGATGCTG	CGAGTTTGCTCTCGTTCTCC
*JmjCA*	TTCCGAATAGCATCCAAAGG	CCGGATCAAATAGCACCACT
*JmjD6*	CAGTTTGCTGGGGAGAGAAG	TGGTTCCTAGAGGGTCGATG
*KDM2*	TGTGTGGGAGAGTCTGGTGA	ATGCCAAAGGACCTGACAGT
*Jumonji* orthologue		
*Jarid 1c*	TCGCAGTGGATGTGGATAAA	ACCTAGCAAGCTGGTCCAAA
*Jumonji 1b*	CCCAGAACACCTGAACCACT	CTGTCCCAGCACTCACTGAA
*Jumonji 4*	CAGCACAACCGAAGGAAGAT	AGCCGCAAGGAGTCTCATAA
*Jumonji 5*	CCTGGACAAATACCAGAAGGAG	ATCTAGACCGTCGTTGTGTAGGAC
*Jumonji 6*	GGTTGGAGGTCAGCTTTCAG	CTGGGCAGTTCATCCATTCT
*Protein Jumonji*	CCGAGAGCCTAATGACGAAG	GGCACAATGACCTTGACCTT
Reference gene		
*EFα*	ACCACCCTGGTGAGATCAAG	ACGACGATCGCATTTCTCTT

## Data Availability

The data presented in this study are available from the corresponding author on reasonable request.
